# The Protective Effect of Glibenclamide in a Model of Hemorrhagic Encephalopathy of Prematurity

**DOI:** 10.3390/brainsci3010215

**Published:** 2013-03-07

**Authors:** Cigdem Tosun, Michael T. Koltz, David B. Kurland, Hina Ijaz, Melda Gurakar, Gary Schwartzbauer, Turhan Coksaygan, Svetlana Ivanova, Volodymyr Gerzanich, J. Marc Simard

**Affiliations:** 1 Department of Neurosurgery, University of Maryland School of Medicine, Baltimore, MD 21201, USA; E-Mails: cigdemtosun@gmail.com (C.T.); mtkoltz@gmail.com (M.T.K.); kurland.davidb@gmail.com (D.B.K.); hinaijaz92@gmail.com (H.I.); meldagurakar@hotmail.com (M.G.); gschwartzbauer@smail.umaryland.edu (G.S.); sveta0652@yahoo.com (S.I.); vgerzanich@smail.umaryland.edu (V.G.); 2 Department of Pathology, University of Maryland School of Medicine, Baltimore, MD 21201, USA; E-Mail: tcoksaygan@vetmed.umaryland.edu; 3 Department of Physiology, University of Maryland School of Medicine, Baltimore, MD 21201, USA

**Keywords:** encephalopathy of prematurity, germinal matrix hemorrhage, intraventricular hemorrhage, choroid plexus hemorrhage, sulfonylurea receptor 1, glibenclamide

## Abstract

We studied a model of hemorrhagic encephalopathy of prematurity (EP) that closely recapitulates findings in humans with hemorrhagic EP. This model involves tandem insults of 20 min intrauterine ischemia (IUI) plus an episode of elevated venous pressure induced by intraperitoneal glycerol on post-natal day (P) 0. We examined Sur1 expression, which is upregulated after focal ischemia but has not been studied after brief global ischemia including IUI. We found that 20 min IUI resulted in robust upregulation of Sur1 in periventricular microvessels and tissues. We studied tandem insult pups from untreated or vehicle-treated dams (TI-CTR), and tandem insult pups from dams administered a low-dose, non-hypoglycemogenic infusion of the Sur1 blocker, glibenclamide, for 1 week after IUI (TI-GLIB). Compared to pups from the TI-CTR group, pups from the TI-GLIB group had significantly fewer and less severe hemorrhages on P1, performed significantly better on the beam walk and accelerating Rotarod on P35 and in tests of thigmotaxis and rapid learning on P35–49, and had significantly greater body and brain weights at P52. We conclude that low-dose glibenclamide administered to the mother at the end of pregnancy protects pups subjected to IUI from post-natal events of elevated venous pressure and its consequences.

## 1. Introduction

The term “encephalopathy of prematurity” (EP) encompasses non-hemorrhagic lesions including cystic and non-cystic periventricular leukomalacia (PVL), hemorrhagic lesions including choroid plexus, germinal matrix and other periventricular hemorrhages that may extend as intraventricular hemorrhages (IVH), various neuronal, axonal and oligodendrocyte pathologies, and hydrocephalus [1,2,3,4,5,6,7]. Infants who survive exhibit cognitive, behavioral and motor (cerebral palsy) abnormalities that persist for life, at immeasurable cost to themselves, their families and society [8,9,10,11,12].

The most severe forms of EP involve hemorrhages that originate in periventricular tissues and extend as IVH. The principal factor determining outcome in these patients is the magnitude of the intracranial hemorrhage [13,14,15]. Hemorrhages not only destroy tissues directly, but blood is toxic to periventricular tissues [16,17], initiating oxidative/inflammatory responses that cause widespread “bystander” injury, especially to white matter, which is highly susceptible to free radical damage.

Analysis of clinical cases of hemorrhagic EP has shown a multifactorial etiology, with three risk factors being especially prominent: (1) prematurity, (2) *in utero* or perinatal ischemia/hypoxia, and (3) early postnatal mechanical ventilation [18,19,20]. Prematurity is associated with immaturity of periventricular vascular structures in both animals [21,22,23] and humans [24,25,26,27,28], which correlates with ongoing angiogenic activity [20,29], and which renders these vessels selectively vulnerable to rupture. Ischemia/hypoxia alters immature periventricular vessels preferentially [30], rendering them yet more vulnerable to rupture. Either condition alone—vascular immaturity or changes due to ischemia/hypoxia, or both, can result in periventricular hemorrhage during periods of elevated venous pressure, as can occur with mechanical ventilation. Veins are the source of most periventricular hemorrhages [31,32], and most hemorrhages occur postnatally, with the onset of bleeding corresponding to the start of mechanical ventilation [33,34]. Mechanical ventilation can increase the pressure in thoracic veins, with the elevated pressure being transmitted to cerebral veins via valveless jugular veins [35]. Thus, ventilation-induced episodes of elevated venous pressure can rupture periventricular veins that are weak due to vascular immaturity or ischemia/hypoxia, leading to hemorrhage within the choroid plexus [2,18,36,37,38,39,40] or in periventricular regions, including the germinal matrix [36,40], and extending as IVH. 

Given these clinical observations, we recently developed a “tandem insult” rat model of hemorrhagic EP, consisting first of 20 min of *in utero* ischemia (IUI) at the “premature time” of E19, 2 days before term, followed by an episode of elevated venous pressure induced by intraperitoneal glycerol 6 h after birth [41]. Neither insult by itself is especially harmful, but in combination, these tandem insults suffered in the perinatal period result in choroid plexus and periventricular hemorrhages involving the subventricular zone (rat equivalent of germinal matrix), hippocampus and white matter, significant developmental delay, and significant vestibulomotor and cognitive abnormalities in young adult rats [41]. Hemorrhages in this model are due to rupture of post-capillary venules [41]. This combination of findings in the animal model recapitulates many of the pathological and neurological observations made in humans with hemorrhagic EP.

Sulfonylurea receptor 1 (Sur1) has been found to be upregulated after focal cerebral ischemia lasting 2 h or more, both in humans and in animal models [42,43], as well as in premature infants with or at risk for germinal matrix hemorrhage [44]. However, Sur1 expression has not been examined in any model of global ischemia, including brief IUI. Blockade of Sur1 has been found to be protective after focal cerebral ischemia, both in humans and in animal models [42,43,45], but the effect of Sur1 blockade has not been examined in any model of global intrauterine ischemia. Here, we evaluated Sur1 expression after IUI, and we evaluated the effect of administering the Sur1 blocker, glibenclamide, to the mother after IUI. Specifically, we studied the effects of drug treatment on hemorrhages occurring shortly after birth, and on neurological function assessed several weeks after birth in pups subjected to tandem perinatal insults.

## 2. Results and Discussion

### 2.1. Sur1 Expression Following IUI

**Figure 1:**
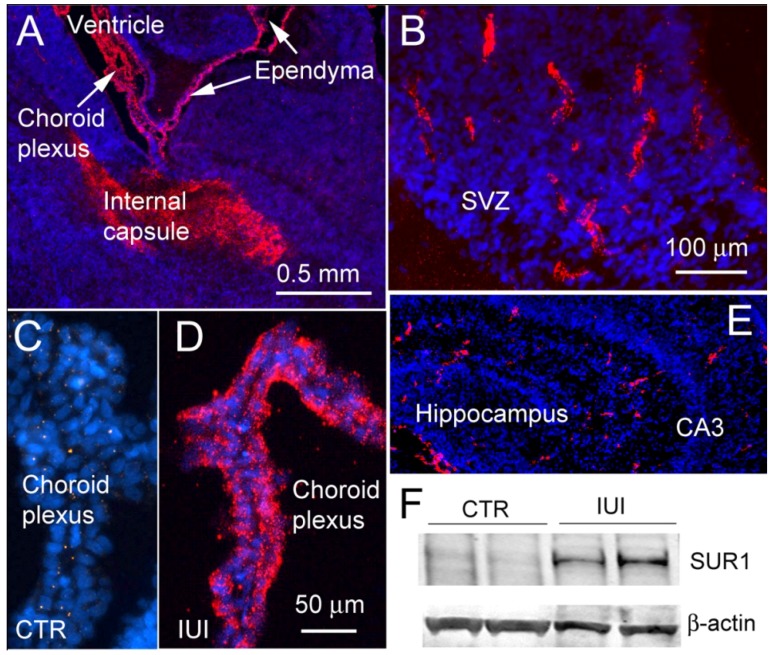
Sulfonylurea receptor 1 (Sur1) is upregulated following 20 min of intrauterine ischemia (IUI). (**A**–**E**): coronal brain sections 24 h after 20 min of IUI (**A**,**B**,**D**,**E**) or from an uninjured sham control (**C**) immunolabeled for Sur1, showing prominent Sur1 expression in choroid plexus, ependyma and internal capsule (**A**,**D**), in the subventricular zone (SVZ) (**B**), and in the hippocampus (**E**) following IUI, but not in the control (**C**); the images shown are representative of data from 10 brains after IUI and 3 controls. (**F**): Immunoblots for Sur1 of periventricular tissues 24 h after 20 min of IUI or from uninjured controls (CTR), as indicated; each lane represents a different brain; β-actin used as a loading control.

We studied the brains of fetuses 24 h after they had been subjected to 20 min of IUI, and compared them to control brains from sham operated E19 fetuses not subjected to IUI. Sur1 expression was examined using immunohistochemistry and immunoblot. Sur1 protein expression was prominent 24 h after IUI, with upregulation found in the choroid plexus, the ependymal lining of the lateral ventricles, and in periventricular tissues, including the internal capsule, the subventricular zone (SVZ), the hippocampus and corpus callosum (Figure 1A,B,D,E). By comparison, Sur1 expression was negligible in control tissues (Figure 1C). Upregulation following IUI was especially prominent in elongated structures consistent with microvessels (Figure 1B,E), as previously reported for models of focal ischemia [46]. Upregulation of Sur1 protein 24 h after a 20 min episode of IUI was confirmed with immunoblots of periventricular tissues; minimal Sur1 protein was detected in tissues from controls not subjected to IUI, compared to a strong single band 24 h after IUI (Figure 1F). Overall, the finding of Sur1 upregulation 24 h after 20 min of global ischemia was consistent with previous observations of prominent upregulation of Sur1 in models of focal ischemia, with both permanent and temporary occlusion [43].

### 2.2. Intravenous Pressure and Hemorrhage

**Figure 2:**
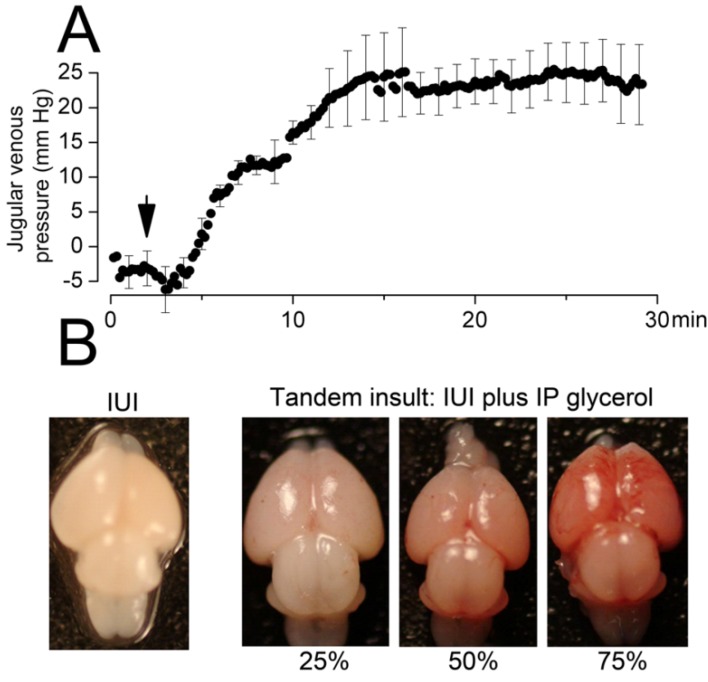
Intraperitoneal glycerol elevates jugular venous pressure and causes brain hemorrhages after intrauterine ischemia (IUI). (**A**) jugular venous pressure as a function of time after injection of 50% glycerol (13 μL/g IP) (arrow); the values shown are the means ± S.E. for measurements in 3 rat pups that were 2 weeks of age (to facilitate catheterization of the jugular vein). (**B**) images of the dorsal surface of brains from rat pups on P1 that had been subjected to IUI 3 days previously (on E19), without IP injection of glycerol (IUI only), or 24 h after injection of 25%, 50% or 75% glycerol (13 μL/g IP); all animals underwent transcardial perfusion with normal saline prior to harvesting the brain; the images shown are representative of triplicate experiments.

In our previous report describing the tandem insult model [41], we showed that an IP injection of 50% glycerol (13 μL/g) was associated with a rise in intrathoracic pressure. We hypothesized that the rise in intrathoracic pressure would be associated with a rise in venous pressure, which would be transmitted to periventricular veins weakened by IUI. Here, we examined this hypothesis directly by measuring jugular venous pressure. An IP injection of 50% glycerol (13 μL/g IP) was associated with a significant increase in jugular venous pressure, which rose from −5 to +25 mm Hg over the course of 15 min (Figure 2A).

**Figure 3:**
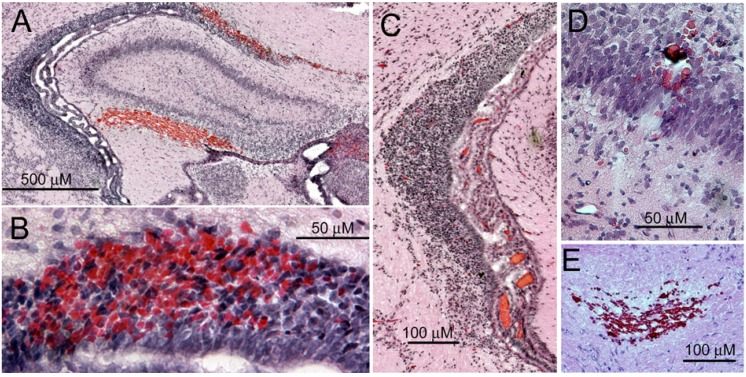
Tandem insults of intrauterine ischemia (IUI) and postnatal elevation in venous pressure cause periventricular hemorrhages. (**A**–**E**) Hematoxylin and eosin (H & E)-stained sections of brains from three rat pups 24 h after the second of 2 tandem insults showing hemorrhages involving white matter near the hippocampus (**A**), the subventricular zone (**B**), the choroid plexus (**C**), the CA3 region of the hippocampus (**D**) and the internal capsule (**E**); all animals underwent transcardial perfusion with normal saline to remove intravascular blood and leave only extravasated blood; the images shown are representative of data from 10 brains after tandem insults.

The concentration of glycerol used for the IP injection had a pronounced effect on the severity of the hemorrhages observed in pups that had been subjected to 20 min of IUI. Keeping the volume of injection constant (13 μL/g, IP), we compared the effect of 25%, 50% and 75% glycerol. In pups subjected to IUI, the use of 75% glycerol resulted in extensive hemorrhages that typically were fatal, whereas 50% glycerol yielded modest periventricular hemorrhages and an acceptable mortality rate (Figure 2B; details on mortality below). In the absence of IUI, 50% glycerol produced no hemorrhages [41], but with IUI, 50% glycerol produced hemorrhages in periventricular regions including choroid plexus, hippocampus, SVZ and corpus callosum (Figure 3A–E). All subsequent experiments were performed with the tandem insults of 20 min IUI and post-natal IP injection of 50% glycerol, 13 μL/g IP.

### 2.3. Serum Glucose, Mortality, Periventricular Hemorrhages and ED-1

**Figure 4:**
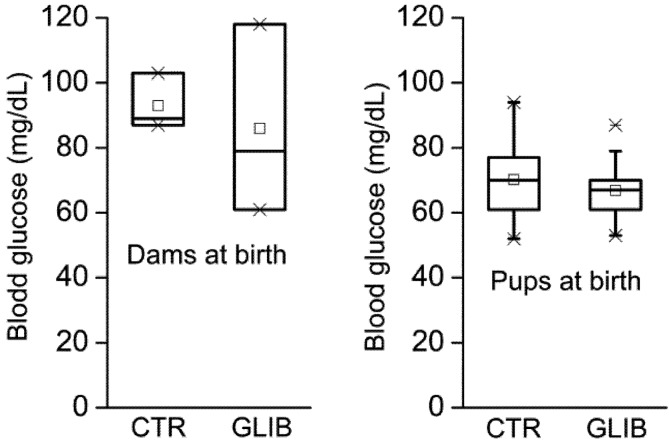
Prenatal low dose glibenclamide infusion does not lower serum glucose. (**A**) values of serum glucose in control dams (CTR) and dams administered low-dose glibenclamide infusion for 2 days (GLIB); 6 dams per group. (**B**) values of serum glucose in control pups (CTR) and pups whose mother had been administered low-dose glibenclamide infusion for 2 days (GLIB); 11 or 13 pups per group; all measurements made shortly after birth. Box plots: large box, 25th and 75th percentiles; whiskers, 5th and 95th percentile; ×, 1st and 99th percentiles; -, max and min; horizontal line, median; small square, mean.

Blockade of Sur1 has been found to be protective after focal cerebral ischemia, both in humans and in animal models [42,43,45]. However, the effect of Sur1 blockade has not been examined in any model of global intrauterine ischemia. In previous experiments on focal ischemia in adult rats, in which drug was delivered directly to the affected organism, we found that an infusion of 200 ng/h of glibenclamide (in addition to a loading dose of 10 μg/kg) was associated with significant protection [43]. Here, we planned to administer glibenclamide to the pregnant dam, necessitating transplacental passage to the affected organism. In the rat, glibenclamide crosses the placental barrier, with the ratio of drug in fetal tissue to that in maternal blood being 0.535 ± 0.068 [47]. We compensated for the indirect delivery to the fetus by administering 400 ng/h (in addition to a loading dose of 10 μg/kg) to the dam. Infusions of this dose of glibenclamide had no significant effect on levels of serum glucose in either the dams at the time of giving birth, or on the pups at birth (Figure 4).

**Figure 5:**
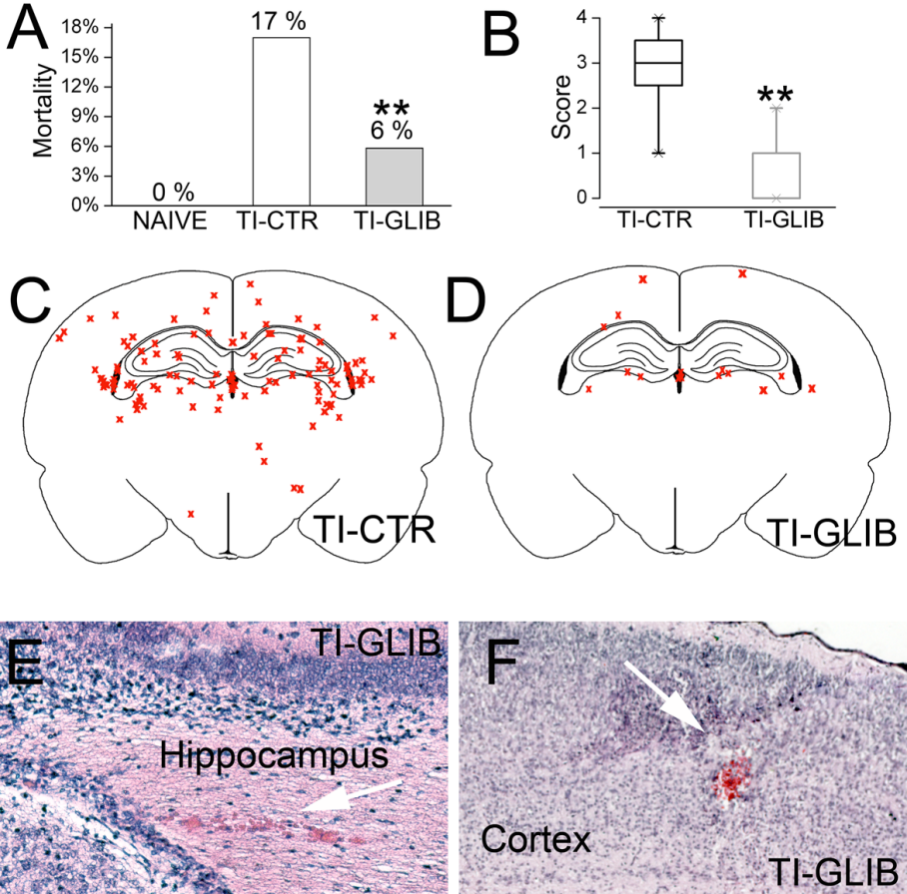
Prenatal low dose glibenclamide infusion reduces mortality, and the incidence and severity of periventricular hemorrhages following tandem insults (TI). (**A**) mortality at 30 h in pups subjected to no injury (naïve), or in pups subjected to TI whose mother was administered no treatment or vehicle treatment (TI-CTR), or in pups subjected to TI whose mother was administered low dose glibenclamide infusion after IUI (TI-GLIB); data from 28 naïve, 72 TI-CTR and 84 TI-GLIB pups, respectively; ** *p* < 0.01. (**B**) hemorrhage scores (see Methods for definitions) at 30 h in TI-CTR pups (*n* = 16) and TI-GLIB pups (*n* = 14); box plot symbols as in Figure 4; note medians are 3 *vs.* 1 for TI-CTR *vs.* TI-GLIB, respectively; ** *p* < 0.01. (**C**,**D**) Distribution of hemorrhages at 30 h in TI-CTR pups (*n* = 16) (**C**) and TI-GLIB pups (*n* = 14) (**D**). (**E**,**F**) H & E-stained sections of brains from two TI-GLIB pups 24 h after TI showing small hemorrhages (arrows) involving white matter near the hippocampus (**E**) and the cortex (**F**); the images shown are representative of data from 10 brains from TI-GLIB pups.

We compared mortality at 30 h in 3 groups: pups that had not been subjected to any insult or exposed to any treatment (naïve), tandem insult pups from untreated or vehicle-treated mothers (TI-CTR), and tandem insult pups from mothers that received glibenclamide after IUI (TI-GLIB). Mortality was nil in the naïve group *versus* 17% in pups from the TI-CTR group [41]. By comparison, mortality was 6% (5/79) in pups from the TI-GLIB group (Figure 5A).

We compared brain hemorrhages 24 h after the second insult in pups from the TI-CTR group *versus* the TI-GLIB group. H & E-stained coronal sections were scored by 3 blinded investigators to assess the severity and distribution of brain hemorrhages. Data were compiled from 16 and 14 rats in the TI-CTR and TI-GLIB groups, respectively. Compared to controls, glibenclamide treatment of the dam was associated with a dramatically reduced severity and incidence of brain hemorrhages 24 h after the second insult (Figure 5B–D). Although there were small hemorrhages near the lateral ventricles in pups from the TI-GLIB group (Figure 5E,F), the majority of the hippocampus and developing cortex typically was spared in these pups.

**Figure 6:**
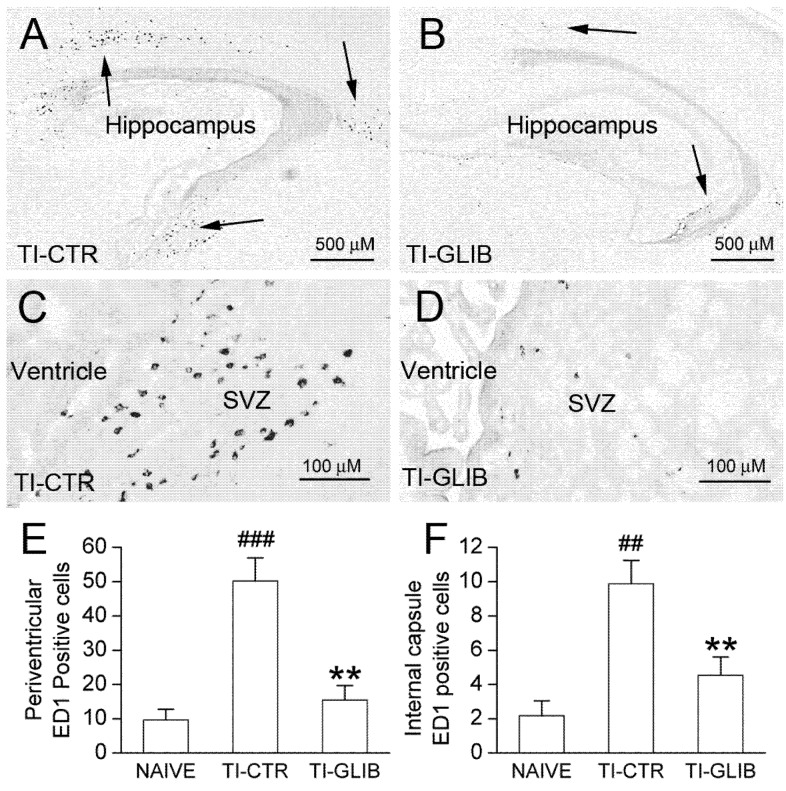
Prenatal low dose glibenclamide infusion reduces the number of ED-1 positive cells following tandem insults. (**A**–**D**) low power views of the hippocampus (**A**,**B**) and high power views of the subventricular zone (SVZ) (**C**,**D**) immunolabeled for ED-1 in pups subjected to TI whose mother was administered no treatment or vehicle treatment (TI-CTR) (**A**,**C**), or in pups subjected to TI whose mother was administered low dose glibenclamide infusion after IUI (TI-GLIB) (**B**,**D**); arrows in (**A**) and (**B**) point to groups of ED-1-positive cells. (**E**,**F**) counts of ED-1 positive cells in periventricular regions (**E**) and in the internal capsule (**F**) in 3 groups of rats, as indicated; ^##^ and ^###^, *p* < 0.01 and *p* < 0.001, respectively, comparing naïve and TI-CTR; ** *p* < 0.01 comparing TI-CTR and TI-GLIB.

In adjacent sections of the same tissues used to evaluate hemorrhages, we also examined ED-1 (rat homologue of CD68), which identifies activated microglia, macrophages and monocytes. ED-1-positive cells in periventricular tissues and the internal capsule were examined. Visual inspection of representative sections showed that ED-1-positive cells were scarcer in tissues from pups in the TI-GLIB group (Figure 6A–D). This observation was confirmed by counts of ED-1-positive cells in both regions, which showed significant elevations in tissues from TI-CTR pups compared to naïve, and significantly fewer cells in tissues from TI-GLIB compared to TI-CTR (Figure 6E,F).

### 2.4. Functional Outcomes

For the neurological functional tests (beam walk, accelerating Rotarod and the Morris water maze (MWM) paradigms), we compared the performances in three groups of rats: naïve, TI-CTR and TI-GLIB.

#### 2.4.1. Beam Walk

**Figure 7:**
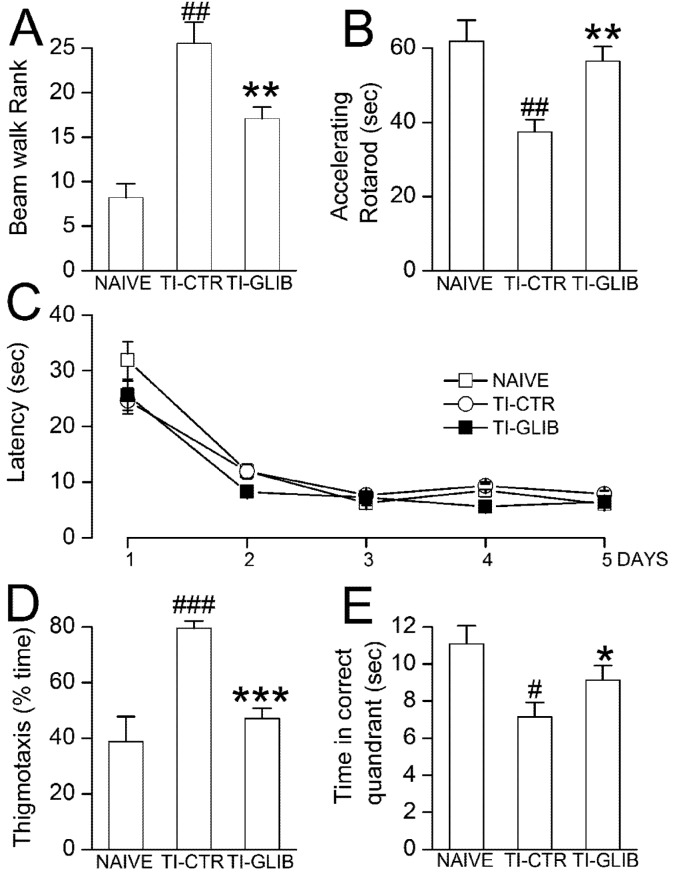
Prenatal low dose glibenclamide infusion improves vestibulomotor and cognitive function following tandem insults (TI). (**A**–**E**) performance at P35 on the beam walk test (**A**), at P35 on the accelerating Rotarod (**B**), at P37–41 on incremental spatial learning (**C**), at P35 on thigmotaxis (**D**), and at P49 on rapid spatial learning (**E**), in pups subjected to no injury (naïve), or in pups subjected to TI whose mother was administered vehicle (TI-CTR), or in pups subjected to TI whose mother was administered low dose glibenclamide infusion after IUI (TI-GLIB); data from 21 naïve, 28 TI-CTR and 28 TI-GLIB pups, respectively; ^##^ and ^###^, *p* < 0.01 and *p* < 0.001, respectively, comparing naïve and TI-CTR; *, ** and ***, *p* < 0.05, *p* < 0.01 and *p* < 0.001, respectively, comparing TI-CTR and TI-GLIB.

On P35, rats in the three groups were tested on the beam walk test. Rats in the TI-CTR group performed poorly compared to rats from the naïve group (Figure 7A). Rats in the TI-GLIB group showed significantly better performance than those in the TI-CTR group, although their performance did not reach the levels of naïve rats.

#### 2.4.2. Accelerating Rotarod

On P35, rats in the three groups were tested using an accelerating Rotarod protocol. Each rat was tested in 3 separate trials, with the best time taken for statistical analysis. Rats from the TI-CTR group performed poorly compared to rats from the naïve group. Rats from the naïve group were able to remain on the apparatus for 61.9 ± 5.6 s, whereas rats from the TI-CTR group remained only 38.5 ± 2.9 s before falling off (Figure 6B). By contrast, the average latency in the TI-GLIB group was 51.3 ± 2.9 s, which was significantly better than in the TI-CTR group, and not different compared to the naïve group (Figure 7B). 

These findings on beam walk and on the accelerating Rotarod indicate that tandem perinatal insults result in significant deficits in complex vestibulomotor function [41], and that glibenclamide treatment of the mother at the end of pregnancy helped to preserve the performance of pups subjected to tandem insults when tested later as young adults.

#### 2.4.3. Thigmotaxis

On P35, rats in three groups began testing in the MWM. During vision testing, there were no differences in average swimming speed between groups (*p* > 0.05; data not shown). All rats eventually reached the visible platform. However, during the first trial of vision testing, rats in the TI-CTR group took longer to reach the platform, compared to rats in the naïve group. The difference in latencies to reach the target during this trial was accounted for by a significantly longer time spent swimming around the periphery of the pool, *i.e.*, thigmotaxis. Rats in the TI-CTR group spent twice the time in thigmotaxis during Trial 1 of Vision testing, compared to rats in the naïve group (Figure 7D). The high incidence of thigmotaxis is consistent with tandem insults producing an abnormal state of anxiety when the rats are introduced to a novel environment [48]. By contrast, rats from the TI-GLIB group exhibited significantly less thigmotaxis than their untreated TI counterparts, with percent times that were significantly less than those of the TI-CTR group, and that were comparable to those of the naïve group (Figure 7D).

#### 2.4.4. Incremental Learning

After the vision test, rats were trained to find a hidden platform kept in a constant location. No differences were observed during this period of ‘incremental learning’, and rats in all groups were able to learn the location of the platform, with performance reaching steady-state for all rats by day 3 (Figure 7C). On P42, one day following the fifth and final day of training, a “memory probe” was performed on each rat, and the total time spent in the correct quadrant, the one that had contained the platform, was measured. Rats in all groups exhibited the correct preference, spending longer than chance (>25% of 60 s) in the quadrant that had contained the platform (data not shown). These data suggested that the ability for incremental acquisition of spatial memory during successive trials was intact, even in rats that had been subjected to tandem insults.

#### 2.4.5. Rapid Learning Task

On P49, an additional MWM test was used to assess a rat’s ability to rapidly learn a new platform location, which is a hippocampus-specific task [49,50,51,52]. During the rapid learning task, each rat was given a single acquisition trial, which was followed by a memory probe after a 30 min interval. For this experiment, % time spent in the correct quadrant was calculated for the first 30 s of the memory probe because after this, rats tended to give up in their search. Rats from naïve group showed a preference for the new quadrant (>25% of the first 30 s), whereas rats from the TI-CTR group showed no such preference (Figure 7E). By contrast, rats from the TI-GLIB group showed significantly better performances in the rapid learning task, exhibiting a preference for the new correct quadrant, similar to but not reaching the level in the naïve group (Figure 7E).

These findings indicated that tandem perinatal insults result in significant deficits in complex learning, specifically related to the hippocampus [41], and that glibenclamide treatment of the mother at the end of pregnancy helps to preserve the performance of pups subjected to tandem insults when they are tested later in life in this complex learning task.

### 2.5. Developmental Delay

**Figure 8:**
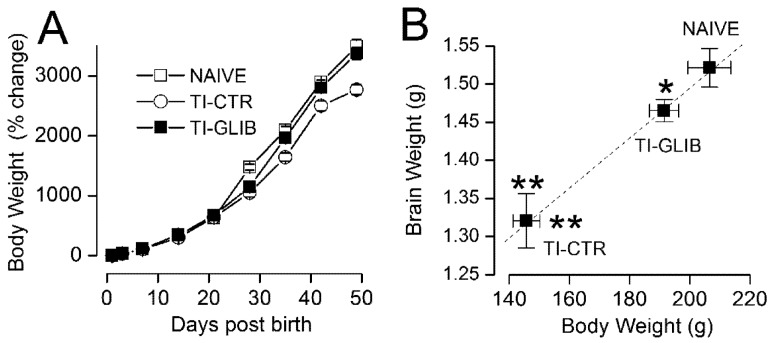
Prenatal low dose glibenclamide infusion reduces developmental delay following tandem insults (TI). (**A**,**B**) Percent gain in body weight (means ± S.E.) as a function of time (**A**), and body weight *versus* brain weight (means ± S.E.) at P52 (**B**) in pups subjected to no injury (naïve), or in pups subjected to TI whose mother was administered vehicle (TI-CTR), or in pups subjected to TI whose mother was administered low dose glibenclamide infusion after IUI (TI-GLIB); data from 12 naïve, 10 TI-CTR and 22 TI-GLIB pups, respectively; ** *p* < 0.01.

Rats were weighed at periodic intervals during development. When measured as % change, rats in the TI-CTR group lagged behind those in the naïve group (Figure 8A). By contrast, those in the TI-GLIB group exhibited better growth, which was indistinguishable from that in the naïve group.

At the time of sacrifice (P52), body and brain weights were significantly lower in rats from the TI-CTR group, compared to naïve, consistent with marked developmental delay associated with the tandem insults [41]. However, pups from TI-GLIB groups were similar to those in the naïve group; there was no significant difference in body weight between the 2 groups, and only a small, albeit significant, difference in brain weight (Figure 8B). These findings suggested that glibenclamide had largely preserved normal somatic and brain development.

Examination of H & E-stained sections of these brains revealed no hydrocephalus or other apparent abnormality in the TI-CTR group, compared to the naïve or TI-GLIB groups. More detailed immunohistochemical analyses will be needed to determine the underlying neuropathological correlate(s) of the functional abnormalities identified in the TI-CTR group.

### 2.6. Discussion

The three principal findings of the present study are: (1) Sur1 is upregulated in periventricular tissues of rats after brief IUI, similar to our findings in premature infants [44]; (2) a low-dose glibenclamide infusion administered to the mother at the end of pregnancy protects pups subjected to the tandem insults of intrauterine ischemia and post-natal elevated venous pressure; (3) the dose of glibenclamide required for protection does not significantly lower serum glucose in either the mothers or the pups. The benefit of glibenclamide was realized early on, in the form of reduced brain hemorrhages secondary to elevated venous pressure and, because of this early protection, the benefit of treatment also was realized later in life, when as young adults, the rats were tested for performance on complex vestibulomotor and complex spatial learning tasks. Arguably, the most notable finding of the present study was that glibenclamide treatment of the mother significantly attenuated developmental delay of the pups and preserved their brain and body mass. Other antenatal treatments with significant efficacy in animal models of periventricular hemorrhage include the selective cyclooxygenase (Cox) 2 inhibitor, celecoxib [29] and glucocorticoids [28,53,54], although antenatal glucocorticoids may have adverse effects on brain function and fetal growth [55,56].

The effect of glibenclamide previously was examined in a rat pup model of neonatal ischemia/hypoxia induced by unilateral carotid ligation plus 2- or 2.5-h exposure (“moderate” and “severe” insults, respectively) to 8% O_2_ in 10-day old pups (modified Rice-Vannucci model) [57]. Glibenclamide (10 μg/kg IP) was administered first shortly after the insult and a second time at 24 h. Brain edema, infarct volume and brain tissue loss were not changed by drug. However, in the moderate injury group, glibenclamide was associated with improvements of motor function in the foot-fault test and in postural reflex test at 3 weeks after ischemic/hypoxic brain injury. The smaller benefit of glibenclamide reported in the Zhou *et al.* [57] study, compared to the present study, may have been due to a more severe ischemic/hypoxic injury than what we studied here. Alternatively, the smaller benefit of glibenclamide may have been due to the amount and manner of drug administered, *i.e.*, to the pharmacokinetics and selectivity of glibenclamide in the rat. The experience in our laboratory [43] and that of others [58] is that a continuous low-dose infusion is important for maintaining constant occupancy of Sur1, for avoiding the off-target Sur2 effect of vasoconstriction [59,60], and for obtaining the most beneficial effect, whereas episodic injections yield positive, albeit less favorable results [61,62]. Here, beginning immediately after IUI, fetuses were exposed continuously to a low dose of glibenclamide while *in utero*, at a concentration half of that in the mother’s serum (ratio of drug in fetal rat tissue to that in maternal blood, 0.535 ± 0.068 [47]). Although the mother continued to receive drug while nursing, it is doubtful that the newborn pups continued to receive glibenclamide, as the transfer of glibenclamide via breast milk is negligible [63,64]. 

Several animal models of periventricular hemorrhage in neonates have been reported [65]. Direct infusion of blood [16] or of collagenase to induce bleeding [66,67,68] reaffirms the toxic effects of blood products on immature periventricular cells [17]. Models intended to shed light on biological mechanisms responsible for hemorrhage generally incorporate a vascular insult of some sort that is superimposed on a predisposing condition such as prematurity [69,70,71] or IUI [41]. The most frequently utilized vascular insult is an IP injection of glycerol. Glycerol is an osmotic agent long said to cause hemorrhage due to a reduction in intracranial pressure (ICP) [70,72], even though the physiological grounds linking a reduction in ICP to the induction of hemorrhage are dubious. Recently, we showed that IP glycerol also causes an increase in intrathoracic pressure [41], and here we show that it causes an increase in jugular venous pressure (Figure 2), which accounts for our previous observation that hemorrhages originate from post-capillary venules [41]. Together, these findings provide a more plausible explanation for the induction of hemorrhage by IP glycerol. Also, these findings support the hypothesis that in humans, mechanical ventilation that may trigger hemorrhage [33,34] is due to elevated venous pressure. Intraperitoneal glycerol has been used to induce hemorrhages in premature rabbits at E29 (full term, 32 days) [70,71,73] and in rats at term that were subjected to IUI on E19 (full term, 21 days) [41]. The rabbit model with IP glycerol has several advantages [71], but suffers from the paucity of validated vestibulomotor and cognitive tests available for this species. Conversely, numerous well validated vestibulomotor and cognitive tests are available for rats, making the rat model with IP glycerol particularly attractive for the study of longer term functional outcomes. 

A combination of mechanisms likely accounts for the fragility of periventricular veins during the several days before term that renders periventricular tissues susceptible to hemorrhage. As detailed in the excellent review by Ballabh *et al.* [20], selective vulnerability has been attributed to an immature basal lamina, incomplete glial support, and poor matrix support, which have been identified in both animals [21,22,23] and humans [24,25,26,27,28]. Immature vessels have prominent ongoing angiogenic activity, and two angiogenic inhibitors, the Cox-2 inhibitor, celecoxib, and the vascular endothelial growth factor receptor 2 inhibitor, ZD6474, reduce this vulnerability [29]. Immature periventricular vessels also are preferentially susceptible to injury by ischemia/hypoxia [30]. As we show here, ischemia/hypoxia results in upregulation of Sur1 in periventricular tissues, and selective blockade of Sur1 using glibenclamide reduces the vulnerability induced by ischemia/hypoxia. In focal ischemia involving both animal models and humans, glibenclamide similarly has been found to protect from ischemia-associated hemorrhage [42,74]. Whether Sur1 is involved here via the Sur1-Trpm4 channel [75] or via some other molecular mechanism has yet to be determined. Notably, exposure of brain endothelium to hypoxic conditions results in upregulation of the transcription factor, specificity protein (Sp) 1 via hypoxia inducible factor 1 [46], and Sp1 upregulation is known to drive the transcriptional expression of both Cox-2 [76] and Sur1 [46,77] in brain endothelium. This shared transcriptional program for Cox-2 and Sur1 in brain endothelium provides an interesting molecular link between the findings of Ballabh *et al.* [29] regarding Cox-2 inhibition and those reported here regarding Sur1 inhibition.

Glibenclamide (United States (US) adopted name, glyburide) is a safe drug approved by government health regulatory agencies worldwide that has been used for more than 3 decades to treat adult onset diabetes mellitus. In the last decade, there has been a growing interest in using glibenclamide to treat gestational diabetes mellitus (GDM) [78,79]. Since 2000, several studies have reported an 80%–85% success rate with the use of glibenclamide in the management of GDM. The use of glibenclamide during pregnancy has given rise to 2 concerns: potential teratogenicity, and fetal hypoglycemia. Some authors have cautioned against possible increases in the risk of preeclampsia, macrosomia, admission to a neonatal intensive care unit and the need for phototherapy with glibenclamide [80,81]. However, animal and human studies assessing the teratogenic effects of oral antidiabetic agents including glibenclamide have yielded equivocal or even negative findings [82], reflecting the fact that the risk of major malformations in infants of mothers with GDM is primarily related to maternal glycemic control, not the specific antidiabetic therapy that is used [83,84]. Some authors have been quite sanguine about the use of glibenclamide for the treatment of GDM, expressing the opinion that “unless future studies refute current data regarding the efficacy and safety of glyburide, we believe that, owing to its ease of administration, convenience and low cost, glyburide will become the first line of medical treatment in patients with GDM within the next few years” [78]. 

The second issue, that of fetal hypoglycemia, is of concern when treating GDM, especially if a large dose of drug is required. At equivalent doses, glibenclamide plasma concentrations are approximately 50% lower in pregnant women than in nonpregnant subjects, potentially necessitating dose escalation [85]. Notably, at the time of delivery, the average umbilical cord/maternal plasma glibenclamide concentration ratio is 0.7 ± 0.4 [85], consistent with some degree of fetal protection from excessive doses. However, the findings presented here as well as in models of focal ischemia [43] indicate that a low, non-hypoglycemogenic dose suffices for protection. Thus, when considering the use of glibenclamide as prophylaxis against the ravages of hemorrhagic EP, it seems unlikely that fetal hypoglycemia would pose a serious safety concern. 

## 3. Experimental Section

*Ethics Statement*. Animal experiments were performed under a protocol approved by the Institutional Animal Care and Use Committee (IACUC) of the University of Maryland, Baltimore. All experiments were performed in accordance with the relevant guidelines and regulations as stipulated in the United States National Institutes of Health Guide for the Care and Use of Laboratory Animals. All efforts were made to minimize the number of animals used and their suffering. 

*Transient Intra-Uterine Ischemia (IUI)*. Timed-pregnant Wistar dams were obtained from Harlan Laboratories (Indianapolis, IN, USA) (embryonic day 1 (E1) corresponds to the day of sperm-positivity following overnight mating). Pregnant dams underwent laparotomy on E19, as previously described [41]. Dams were anesthetized with 3% isoflurane delivered with 75% air + 25% O_2_; anesthesia was maintained with 2% isoflurane for the duration of surgery. Pulse oximetry (Mouse-Ox; STARR Life Sciences Corp., Oakmont, PA, USA) was used to maintain O_2_ saturation 90%–95%. A heating pad was placed beneath the dam to maintain body temperature at ~37 °C. A laparotomy was made, the uterus was externalized and protected with 4 × 4 gauze sponges moistened with normal saline (NS) covered with a sheet of latex to prevent dehydration of the tissues. The uterine and ovarian vasculature was clamped for 20 min to induce transient intrauterine ischemia (IUI) [86] using vascular clamps with low closing pressure (5–15 g/mm^2^; Fine Science Tools, Foster City, CA, USA). Care was taken to clamp both the uterine and ovarian vasculature bilaterally, in order to ensure global ischemia to all pups. Laser Doppler flowmetry (LDF) confirmed the reduction in blood flow [41]. After removing the microvascular clamps, we checked to make sure that the pulse recovered to all fetuses, following which the uterus was re-internalized. In some cases, all but 5 mm of the abdominal incision was closed, with the remaining opening used to inject either vehicle or glibenclamide intraperitoneally (IP) (see below) before the remainder of the laparotomy was closed. In these cases, a mid-scapular incision was made and a mini osmotic pump (Alzet 2001, 1.0 µm/h, Alzet Corp., Cupertino, CA, USA) was implanted subcutaneously for continuous infusion of vehicle or glibenclamide to the dam over the next 7 days. The dam was allowed to recover from anesthesia. The duration of anesthesia was ~35 min. Controls were from dams left untouched (naïve).

*Glibenclamide Treatment of the Mother*. Drug formulation and the preparation of mini-osmotic pumps have been described in detail [87]. Briefly, a stock solution of glibenclamide (#G2539; Sigma, St. Louis, MO, USA) was prepared by placing 25 mg into 10 mL of dimethyl sulfoxide (DMSO). The solution used for the loading dose was made by placing 4 μL of stock solution into 1 mL unbuffered NS. The solution for infusion was made by placing 4 μL of 10 N NaOH into 2.1 mL of NS, then adding 400 µL of stock solution, in that order, to prevent precipitation of drug [87]. Treatment consisted of: (1) administering a single loading dose of glibenclamide (10 μg/kg) or an equivalent volume of vehicle IP immediately before closing the laparotomy; (2) continuous infusion via mini osmotic pump beginning at the end of surgery, resulting in delivery of 400 ng/h or an equivalent volume of vehicle subcutaneously for 1 week. 

*Birth and Post-Natal Insult*. Spontaneous, unaided vaginal delivery usually occurred 2–3 days (E21–22) after IUI. The day of birth is defined as P0. Newborn pups were allowed 6 h undisturbed to bond with the mother. At 6 h, pups received an injection of 50% glycerol (13 μL/g IP) to cause a transient rise in intravenous (IV) pressure (see Results). Care was taken to minimize the time that pups were removed from the mother. 

*Serum Glucose*. Measurements of serum glucose were obtained by bleeding the tip of the tail to obtain a 5 μL sample of blood in dams and pups shortly after birth. Serum glucose was measured using a glucometer (OneTouch, Lifescan, Milpitas, CA, USA).

*Experimental Series and Groups*. We studied 3 experimental series (Table 1). In series 1, pups subjected to IUI alone were harvested 24 h after IUI and the brains were examined for Sur1 upregulation using immunochemistry and immunoblot. In series 2, we studied 3 groups: pups that had not been subjected to any insult or exposed to any treatment (naïve), tandem insult pups from untreated or vehicle-treated dams (TI-CTR), and tandem insult pups from dams that received glibenclamide for 1 week after IUI (TI-GLIB); pups in this series were used to calculate mortality; surviving pups were euthanized on P1, 24 h after the second insult (~30 h after birth); some of the brains from this series were used to determine the incidence and severity of intracranial hemorrhages, and to examine for ED-1 positive cells by immunochemistry; fthese pups were not studied behaviorally. In series 3, we studied 3 groups: naïve, TI-CTR, and TI-GLIB; these groups were weighed periodically and underwent neurofunctional testing (see below) up to P52, at which time brain weights were determined.

**Table 1:** Number of rats in 3 experimental series.

		Number of Dams	Number of Pups
**SERIES 1**	naïve	2	6
	IUI	3	12
**SERIES 2**	naïve	3	28
	TI-CTR	7	72
	TI-GLIB	7	84
**SERIES 3**	naïve	2	21 or 30 *
	TI-CTR	4	28 or 37 *
	TI-GLIB	4	28 or 37 *

IUI, intrauterine ischemia; TI-CTR, tandem insult pups from untreated or vehicle-treated dams; TI-GLIB, tandem insult pups from dams that received glibenclamide; * the larger value applies to the accelerating Rotarod test; the smaller value applies to Morris water maze tests.

Some of the results presented here as TI-CTR (see Figure 5, Figure 6) were reported in a previous paper from this laboratory that first described the tandem insult model [41] and, as noted above, are pooled from dams untreated and from dams administered vehicle after 20 min IUI. Importantly, experiments on vehicle *versus* glibenclamide treatment were performed concurrently by the same investigators, who were blinded to actual treatment.

*Measurement of Jugular Venous Pressure*. Rat pups 2 weeks old (to permit catheterizing the jugular vein) were anesthetized (ketamine, 100 mg/kg, plus xylazene, 10 mg/kg, i.p.) and, using microscopic technique, the probe (1 mm diameter) of the pressure transducer was introduced into the jugular vein. Intravenous pressure was measured using a factory-calibrated system (PhysiolTel Transmitter, model PA-C10; PhysioTel Receiver, model RPC-1; and Dataquest A.R.T. system for acquisition and analysis; Data Sciences International, St. Paul, MN, USA).

*Scoring Brain Hemorrhage*. Pups in series 2 that survived the second insult were euthanized 30 h later. Coronal sections of these brains were stained with H & E and were used to determine the incidence, location and magnitude of brain hemorrhages (the brains of pups that died generally were not suitable for examination). An unbiased scoring system was used for grading hemorrhages: (1) 1 point was assigned for hemorrhage in any of the following 3 locations: choroid plexus, intraventricular and periventricular, with no more than 1 point per location being scored; (2) 1 point was scored if hemorrhage could be observed grossly during cryosectioning. Thus, a score of 0–4 was assigned for each pup. Data were collected by 3 blinded independent investigators, with the final score being the average score of the 3.

*Mortality*. Mortality was determined during the first 30 h after birth from pups in series 2; pups that survived this initial period rarely died later. For groups subjected to TI, mortality was based on the number of pups detected during laparotomy. For pups without injury (naïve), *in utero* counts were not made, and only observed postnatal deaths could be counted.

*Motor Assessment*. Vestibulomotor function was tested using the beam walk task [88,89], and the Rotarod (Series 8, IITC Life Science, Woodland Hills, CA, USA) with an accelerating protocol (starting with 4 rpm and accelerating to 45 rpm in 100 s). Rats were allowed to stay on for a maximum of 180 s. The latency to fall from the wheel was recorded for each trial.

*The Morris Water Maze (MWM)*. The MWM was used to assess spatial learning and memory [90,91]. The device and the specific paradigms used for MWM testing in this laboratory have been described [92]. As an index of open space anxiety-related behavior, we also analyzed thigmotaxis. A rat was considered to be in thigmotaxis if it swam at a distance within 10 cm of the wall of the pool. For each group, the time spent in thigmotaxis was calculated for the first trial of vision testing in the MWM. 

*Immunolabeling*. Pups (P1) were processed to minimize freeze artifact [93]. Brains were perfusion-fixed with 10% neutral buffered formalin. After fixation, brains were cryoprotected using 30% sucrose in PBS for 48 h at 4 °C, or were placed into 70% ethanol prior to paraffin embedding.

Cryosections (20 μm) were collected on slides, blocked in 2% donkey serum with 0.2% Triton X-100 in PBS for 1 h, then incubated overnight at 4 °C with goat anti-Sur1 antibody (1:200; Santa Cruz Biotechnology, Santa Cruz, CA, USA) or mouse anti-ED-1 antibody (1:200; Millipore, Billerica, MA, USA). After several rinses in phosphate buffered saline, slides were incubated for 1 h with fluorescent-labeled secondary antibodies (1:500; Alexa Flour 555 or Alexa Flour 488; Invitrogen, Molecular Probes, Eugene, OR, USA) at room temperature. The sections were coverslipped with polar mounting medium containing antifade reagent and 4’,6-diamidino-2-phenylindole (DAPI; Invitrogen, Eugene, OR, USA). Slides were examined using epifluorescence microscopy (Nikon Eclipse 90i; Nikon Instruments Inc., Melville, NY, USA). Control experiments involved omission of the primary antibody.

*Immunoblot*. The brain was cut into 3 equal pieces, and the center slice that contained the majority of periventricular tissues was used for homogenization. Tissue homogenates were immunoblotted as described [75].

*Data Analysis*. Non-parametric datasets (hemorrhage scores, beam walk scores) were rank-transformed prior to analysis [94]. Statistical analysis was performed using a 1-way ANOVA with Fisher *post-hoc* comparisons. Mortality data were analyzed using a 2 × 2 contingency table and Fisher’s exact test (two-tailed). Significance was accepted if *p* <0.05.

## 4. Conclusion

Low-dose glibenclamide administered to the mother at the end of pregnancy protects pups subjected to IUI from post-natal events of elevated venous pressure and its deleterious consequences on neurological function later in life. Given that Sur1 is upregulated in the germinal matrix of premature infants [44], that glibenclamide already is in use in pregnant women, and that no teratogenic issues have been identified, and that low, non-hypoglycemogenic doses are protective, it seems that a unique opportunity may be at hand to examine the potential benefit of glibenclamide in high risk pregnancies.
